# Co-expression module analysis reveals biological processes, genomic gain, and regulatory mechanisms associated with breast cancer progression

**DOI:** 10.1186/1752-0509-4-74

**Published:** 2010-05-27

**Authors:** Zhiao Shi, Catherine K Derow, Bing Zhang

**Affiliations:** 1 Advanced Computing Center for Research & Education, Vanderbilt University, Nashville, TN 37240, USA; 2 Department of Electrical Engineering and Computer Science, Vanderbilt University, Nashville, TN 37240, USA; 3 Department of Biomedical Informatics, Vanderbilt University School of Medicine, Nashville, TN 37240, USA

## Abstract

**Background:**

Gene expression signatures are typically identified by correlating gene expression patterns to a disease phenotype of interest. However, individual gene-based signatures usually suffer from low reproducibility and interpretability.

**Results:**

We have developed a novel algorithm Iterative Clique Enumeration (ICE) for identifying relatively independent maximal cliques as co-expression modules and a module-based approach to the analysis of gene expression data. Applying this approach on a public breast cancer dataset identified 19 modules whose expression levels were significantly correlated with tumor grade. The correlations were reproducible for 17 modules in an independent breast cancer dataset, and the reproducibility was considerably higher than that based on individual genes or modules identified by other algorithms. Sixteen out of the 17 modules showed significant enrichment in certain Gene Ontology (GO) categories. Specifically, modules related to cell proliferation and immune response were up-regulated in high-grade tumors while those related to cell adhesion was down-regulated. Further analyses showed that transcription factors NYFB, E2F1/E2F3, NRF1, and ELK1 were responsible for the up-regulation of the cell proliferation modules. IRF family and ETS family proteins were responsible for the up-regulation of the immune response modules. Moreover, inhibition of the PPARA signaling pathway may also play an important role in tumor progression. The module without GO enrichment was found to be associated with a potential genomic gain in 8q21-23 in high-grade tumors. The 17-module signature of breast tumor progression clustered patients into subgroups with significantly different relapse-free survival times. Namely, patients with lower cell proliferation and higher cell adhesion levels had significantly lower risk of recurrence, both for all patients (*p *= 0.004) and for those with grade 2 tumors (*p *= 0.017).

**Conclusions:**

The ICE algorithm is effective in identifying relatively independent co-expression modules from gene co-expression networks and the module-based approach illustrated in this study provides a robust, interpretable, and mechanistic characterization of transcriptional changes.

## Background

Large-scale gene expression profiling with microarray platforms is becoming a popular approach to the identification of molecular biomarkers for disease diagnosis, prognosis, and response to treatment. Gene expression signatures are usually identified by correlating gene expression patterns to a disease phenotype of interest. Since the pioneering work on breast cancer [1,2], gene expression signatures have been reported for different diseases. Some of the signatures have been approved by the US Food and Drug Administration for diagnostic assay.

Despite these exciting progresses, individual gene-based signatures suffer from some well-known problems. First, given the large number of genes on the array, high correlation among the genes, small number of samples in a data set, and large variance across patient samples, it is common to see little overlap among gene signatures identified by different research groups for a common clinical outcome [3]. Secondly, because gene-based signatures often fail to put individual genes in a functional context, understanding the biology highlighted by a signature remains a significant challenge[4]. Moreover, because important regulators such as transcription factors are not necessarily regulated at the transcriptional level, gene-based signatures can seldom identify regulatory mechanisms underlying the disease phenotype of interest. As a result, module-based approaches aimed at a more robust and interpretable characterization of transcriptional changes have emerged [4-6].

In contrast to the gene-level analyses, module-based approaches use gene modules as the basic building blocks for analysis. Modules can be defined in a knowledge-driven fashion based on existing knowledge on pathways, biological processes, and protein complexes [5]; they can also be derived in a data-driven fashion by identifying subgroups of genes sharing similar expression pattern across multiple conditions, i.e., co-expression modules [7]. The latter is particularly interesting as it is not limited to or biased towards existing knowledge, and holds the potential to reveal truly novel regulatory mechanisms underlying the dynamic molecular processes underpinning disease[8]. Various studies have demonstrated the significance of examining gene co-expression in addressing biological problems [7,9,10].

Some commonly used methods for analyzing gene co-expression pattern in microarray data include hierarchical clustering [11], K-means clustering [12], and self organizing maps [13]. Generally, these methods are suitable for understanding the global structure of the data but suboptimal for module identification. Recently, various methods for module detection in large-scale networks have been proposed. Some representative methods include the betweenness-based method [14], the modularity optimization method [15], the spectral partitioning method [16], and graph-theoretic approaches relying on cliques and other tightly connected components [17,18]. Clique-based approaches have to date provided one of the most successful ways to consider module overlap, which is an important characteristic of real networks [18]. For example, a gene/protein can have multiple functions and therefore belong to multiple modules or complexes. In fact, clique-based approaches have been successfully applied on the identification of protein complexes from protein interaction networks [18-21]. A clique is a complete sub-network in which all nodes are connected in a pairwise fashion. A clique is maximal if it is not contained in any other clique, and maximum if it is the largest maximal clique in the graph. Clique-based approaches usually start with the identification of all maximal cliques in the network. Because maximal cliques overlap in real networks, algorithms have been developed to merge highly overlapping cliques to facilitate further analysis [18,21].

Applying graph-theoretic approaches on gene expression data requires the construction of gene co-expression network from the data [22]. Usually, gene expression similarity is calculated for each pair of genes, and then a network is constructed by setting a threshold for the pair-wise similarity. Such a network is usually represented as an un-weighted graph, in which each node is a gene and two genes are connected by an edge if their expression similarity level is above a pre-selected threshold. The choice of a threshold can significantly affect the integrity of the network and the co-expression modules derived from it. Various methods have been proposed in an attempt to guide the selection of an appropriate threshold, including those based on statistical analysis [23,24] or network properties [25,26]. Usually, functional similarity derived from Gene Ontology (GO) is used subsequently to evaluate the biological relevance of the selected threshold [25,27].

The application of clique-based approaches on gene co-expression networks is hampered by an extreme degree of overlap among maximal cliques in a network. While allowing overlap is an important advantage of clique-based approaches, excessive overlap carries too much redundant information and makes downstream analysis difficult. Although one may consider a post-processing step to merge highly overlapping cliques [18,21], this is usually impractical for gene co-expression networks because there may exist billions of maximal cliques in a network and it is already a significant challenge just to store these cliques.

In this paper, we propose a clique-based framework for the analysis of gene expression data. An iterative clique enumeration (ICE) algorithm is developed to identify a manageable number of modules that can represent major transcriptional programs encoded in a co-expression network. Downstream analyses on the modules further reveal biological processes and regulatory mechanisms underlying disease phenotypes of interest. Using publicly available human breast cancer gene expression datasets, we demonstrated that the ICE algorithm was able to detect functionally homogeneous co-expression modules, and the detected modules covered a diverse variety of biological processes. We further identified modules whose overall expression levels were correlated with tumor grade, and showed that the correlations were more reproducible in an independent data set than those based on individual genes or modules identified by other algorithms. Next, we illustrated that the module-based approach could reveal important biological processes as well as previously reported and novel regulatory mechanisms underlying breast tumor progression. Finally, we showed that the expression pattern of the modules could cluster patients into subgroups with significantly different relapse-free survival times and provided useful prognostic information that was independent of tumor grade.

## Results and Discussion

### Overview of the co-expression module-based analysis framework

**Figure 1:**
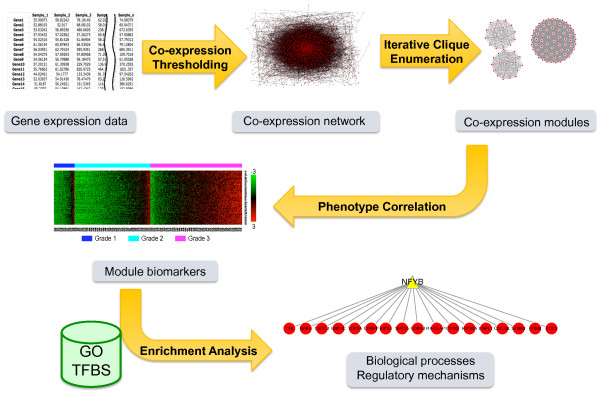
**Schematic overview of the co-expression module-based analysis framework**. GO: Gene Ontology. TFBS: Transcription Factor Binding Sites.

Figure 1 depicts an overview of the co-expression module-based analysis framework. Based on a gene expression data set, a co-expression network is constructed in which each node is a gene and two genes are connected by an edge if their expression similarity level is above a pre-selected threshold. Although we used the Pearson's correlation coefficient for the similarity calculation in this study, other measurements such as the Spearman's correlation coefficient and the mutual information can be equally applied. A knowledge-guided method is employed for threshold selection to ensure the biological relevance of the gene co-expression network. Next, the ICE algorithm developed in this study is used to identify relatively independent maximal cliques as co-expression modules. In contrast to the single gene-based analyses in which individual genes are tested for their correlation to a phenotype of interest (e.g. tumor grade or stage), the module-based approach analyzes modules as units and identifies co-expression modules that are significantly correlated with the phenotype, i.e. potential module biomarkers. Finally, identified modules are queried against gene set databases such as the GO gene sets and Transcription Factor Binding Site (TFBS) gene sets to infer biological processes and regulatory mechanisms underlying the phenotype of interest.

### Knowledge guided co-expression network construction

Microarray gene expression data on two cohorts of breast cancer patients were used for demonstration in this study. Both datasets (GSE2109_breast and GSE2990 [28]) were downloaded from the Gene Expression Omnibus (GEO) database http://www.ncbi.nlm.nih.gov/geo/. GSE2109_breast was generated using the Affymetrix U133 Plus 2.0 Array, while GSE2990 was generated using the Affymetrix U133A Array. Clinical information on patients in each dataset is summarized in Table 1.

**Table 1:** Microarray datasets used in the study

		GSE2109_breast	GSE2990
**Sample size**		351	189

**Pathological stage**	**0**	4	-
	
	**1**	38	-
	
	**2**	129	-
	
	**3**	67	-
	
	**4**	5	-
	
	**NA**	108	-

**Pathological grade**	**1**	31	67
	
	**2**	113	46
	
	**3**	136	59
	
	**NA**	71	17

**Recurrence**	**1**	-	67
	
	**0**	-	120
	
	**NA**	-	2

**Figure 2:**
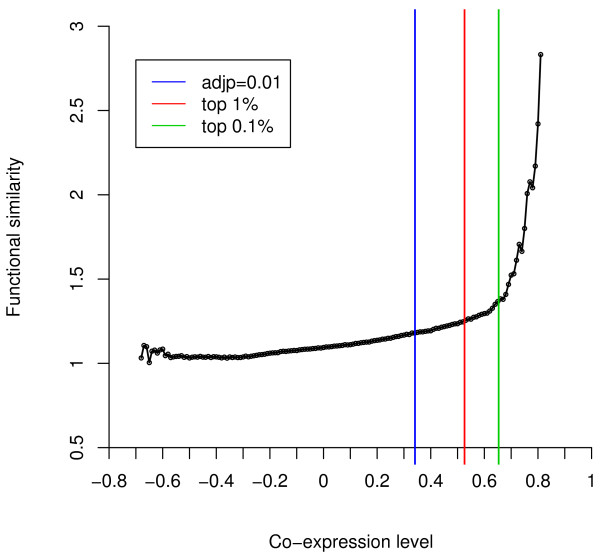
**Functional similarity for gene pairs at different co-expression level**. Co-expressions are binned into 0.1 unit intervals and the average GO semantic similarity for each bin is plotted as an open circle. The three vertical lines correspond to three co-expression levels: Bonferoni corrected *p *value of 0.01 (blue), the top 1% of all correlations (red), and the top 0.1% of all correlations (green).

We used the GSE2109_breast dataset consisting of 351 breast tumor specimens for co-expression network construction. The construction of co-expression network by thresholding is a critical step in the analysis framework. To ensure the biological relevance of constructed network, we used a knowledge-guided method for threshold selection. Figure 2 shows the relationship between co-expression level (as measured by the Pearson's correlation coefficient) and functional similarity (as measured by GO semantic similarity). As shown in the figure, in general, a higher co-expression level corresponds to a higher functional similarity score. Although large positive and negative correlations are both statistically significant, large negative correlations don't correlate with higher functional similarity. We examined different threshold selections corresponding to the Bonferroni adjusted *p *value of 0.01, the top 1% of all correlations, and the top 0.1% of all correlations (Figure 2). No critical functional similarity change was observed at the co-expression level corresponding to the Bonferroni adjusted *p *value of 0.01, indicating that statistical significance couldn't be transferred directly into biological significance. In contrast, a sharp increase in functional similarity was observed above the co-expression level corresponding to the top 0.1% of all correlations. Therefore, we used this threshold (Pearson's correlation coefficient of 0.6533) and constructed a gene co-expression network with 7,819 nodes and 195,928 edges. Because biological networks, including co-expression networks, usually share some important characteristics such as the power law degree distribution and high clustering coefficient [7,29], we further examined these topological characteristics of the constructed network. Indeed, the network showed a power law degree distribution (data not shown) and a high clustering coefficient of 0.52, suggesting its biological relevance.

### Functional homogeneity within and heterogeneity across co-expression modules

The extremely high clustering coefficient of the network indicated a highly modular structure. In the ICE algorithm, we define co-expression modules as subgroups of perfectly interconnected genes, i.e., cliques. A classic maximal clique enumeration algorithm [30] identified 1,306,734,139 maximal cliques of size 10 or more from the network, making existing methods relying on the maximal cliques such as the C-Finder algorithm [18] impractical. Applying the ICE algorithm proposed in this study, we identified 50 maximal cliques as relatively independent co-expression modules. A major concern in the application of clique-based approaches is their computational complexity. However, as gene co-expression networks are sparse with a scale-free distribution, clique-based approaches are applicable on these networks [22]. For the above analysis, it took only 8 minutes and about 128 MB memory for the ICE algorithm to generate the results on a computer with a 2.4 GHz AMD Opteron CPU and 4 GB memory.

Because all genes in a module are highly co-expressed and are likely co-regulated, we expect functional homogeneity within the modules. On the other hand, because overlap between the modules is restrained, we expect the modules to be functionally heterogeneous and to represent relatively independent biological processes and transcriptional regulatory programs. In order to evaluate the functional homogeneity of the modules, we performed the functional category enrichment analysis for the modules against the GO categories. Among the 50 modules, 36 (72%) showed high functional homogeneity, with a Bonferroni-adjusted *p*-value (B-adjp) less than 0.05 in at least one of the GO categories. Moreover, these modules corresponded to a diverse variety of biological processes including metabolic process, immune system process, developmental process, system process, cell cycle, response to stimulus, transport, signal transduction, etc. These results suggest that co-expression modules identified by the ICE algorithm are functionally homogeneous within a module and heterogeneous across modules. Lack of functional enrichment for some of the modules may be attributed to the false discovery of the algorithm, the incompleteness of the GO annotations, or the non-functional relationship among genes in a module (e.g. genomic proximity).

### Modules correlated with breast cancer progression

The modules identified above were dynamically expressed in response to different conditions. Because samples in the dataset included tumor specimens from different stages and grades, we hypothesized that the dynamic expression of some of the modules might correlate with tumor stage or grade. Average expression of all genes in a module was used to represent the overall expression level of the module. We used the non-parametric Jonckheere-Terpstra trend test to evaluate the correlation between the expression levels of the modules and stage or grade. Because we were testing 50 modules at the same time, the *p *values generated by the test were further adjusted using the Benjamini and Hochberg correction [31] to derive False Discovery Rates (FDRs). To our surprise, none of the modules was correlated with tumor stage (FDR > 0.46). On the other hand, 19 out of the 50 modules were significantly correlated with tumor grade (FDR < 0.01), and some of the correlations were extremely significant. For example, module_2 showed an FDR of 3.52e-22. Module_2 is used for illustration in Figure 1. As shown in the heat map, all genes in this module were highly correlated, and their expression levels were drastically increased in grade 3 tumors compared to grade 1 and 2 tumors. These results are consistent with a previous report that different tumor grades are associated with distinct gene expression signatures, while tumor specimens from distinct pathological stages may share remarkable similarity in the expression profiles [32].

### Reproducibility of the module-based biomarkers in independent data set

Potential application of molecular features (e.g. individual genes or modules) as disease biomarkers relies on the reproducibility of the association between molecular features and the disease phenotype in independent patient cohorts. Ideally, if we find a biomarker from one patient cohort, we hope that this biomarker could hold significant in independent cohorts. In this study, we define reproducibility of a biomarker set identified from one patient cohort as the percentage of included biomarkers that hold significant in an independent cohort.

19 modules showed significant correlation with tumor grade in the GSE2109_breast dataset (FDR < 0.01). We calculated the percentage of the correlations that held significant in the independent dataset GSE2990. Despite GSE2990 being generated on a different microarray platform on which only 64% of the genes in the GSE2109_breast dataset were present, 17 out of the 19 modules (89.47%) showed significant correlation with tumor grade (FDR < 0.01) in this independent dataset, indicating high reproducibility of this module-based biomarker set. We also checked the modules for their gene components. The 19 modules identified in the GSE2109_breast dataset comprised 405 genes, among which 371 were included in the 17 reproducible modules (91.60%). When we focused on genes common to both platforms, the 19 modules comprised 352 common genes, among which 330 were included in the 17 reproducible modules (93.75%).

**Figure 3:**
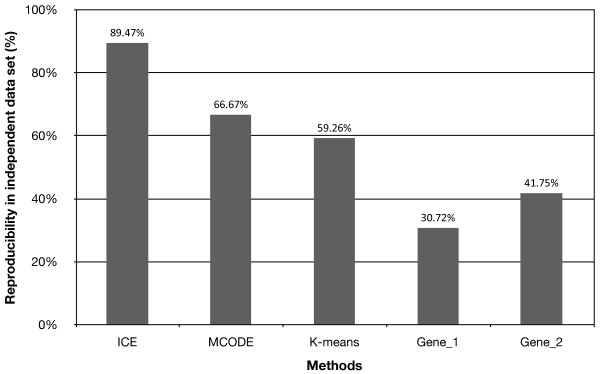
**Reproducibility of grade-correlation in an independent data set**. Modules or genes correlated with tumor grade were identified in the GSE2109 dataset using different methods and tested for reproducibility in the GSE2990 dataset. ICE, MCODE, and K-means are three module-based methods. Gene_1 and Gene_2 are based on individual genes. Gene_1 does not exclude the difference between microarray platforms used for the two datasets, while Gene_2 is based on common genes on the microarray platforms.

As a comparison, we repeated the analysis at the individual gene level. Among the 19,803 genes in the GSE2109_breast dataset, 5,107 showed significant correlation with tumor grade (FDR < 0.01). When tested in GSE2990, only 1,569 genes (30.72%) maintained significant correlation with tumor grade (FDR < 0.01). Some of the inconsistency can be explained by the difference between the array platforms because only 3,758 out of the 5,107 genes were presented in the GSE2990 dataset. Nevertheless, even when we considered only the 3,758 genes, the gene-level reproducibility (41.75%) was still much lower than that for the ICE modules (Figure 3).

We also used the popular partitioning method K-means and the graph-based module identification algorithm MCODE to identify modules from the GSE2109_breast dataset. For the K-means analysis, the number of clusters was arbitrarily set to 50 to match the number of modules identified by ICE. For the MCODE analysis, 37 modules with 10 or more genes were identified. Based on the Jonckheere-Terpstra trend test, 27 K-means modules and 21 MCODE modules were significantly correlated with tumor grade (FDR < 0.01) in the GSE2109_breast dataset. When tested in the GSE2990 dataset, 16 K-means modules (59.26%) and 14 MCODE modules (66.67%) showed significant correlation with tumor grade (FDR < 0.01).

These comparisons demonstrate that module-based biomarker sets are more reproducible than the one based on individual genes when the same selection criterion (FDR < 0.01) is applied. Using more stringent criteria for the selection of gene-based biomarkers may improve the reproducibility of the selected biomarkers [28]. However, with a very stringent criterion, the functional context of selected biomarkers may be lost, making it difficult to understand the underlying biology. For the module-based methods, although the numbers of reproducible modules were comparable for the three methods, the ICE method outperforms both K-means and MCODE with regard to the reproducibility (Figure 3). The higher reproducibility of ICE may be attributed to the knowledge-guided network construction and the stringent requirement imposed by the module definition in the ICE algorithm that ensures high-level of co-expression among all genes in a module. Incorporating GO information in the K-means analysis might help improve its performance. Although MCODE starts with the same network as ICE, high-level of co-expression among all genes in a module is not guaranteed. High-level of co-expression is critical for the inference of co-regulation [8], therefore, the ICE algorithm is more likely to identify truly co-regulated gene sets that are reproducible in independent data sets. On the other hand, owing to its stringent requirement, the ICE algorithm is likely to miss some genes in a module, which will limit its use in applications sensitive to false negatives. However, as long as the core components in a co-expression module can be identified, one should be able to use the modules as biomarkers or for the inference of underlying regulatory mechanisms through enrichment analysis.

### Major biological processes associated with breast tumor progression

**Figure 4:**
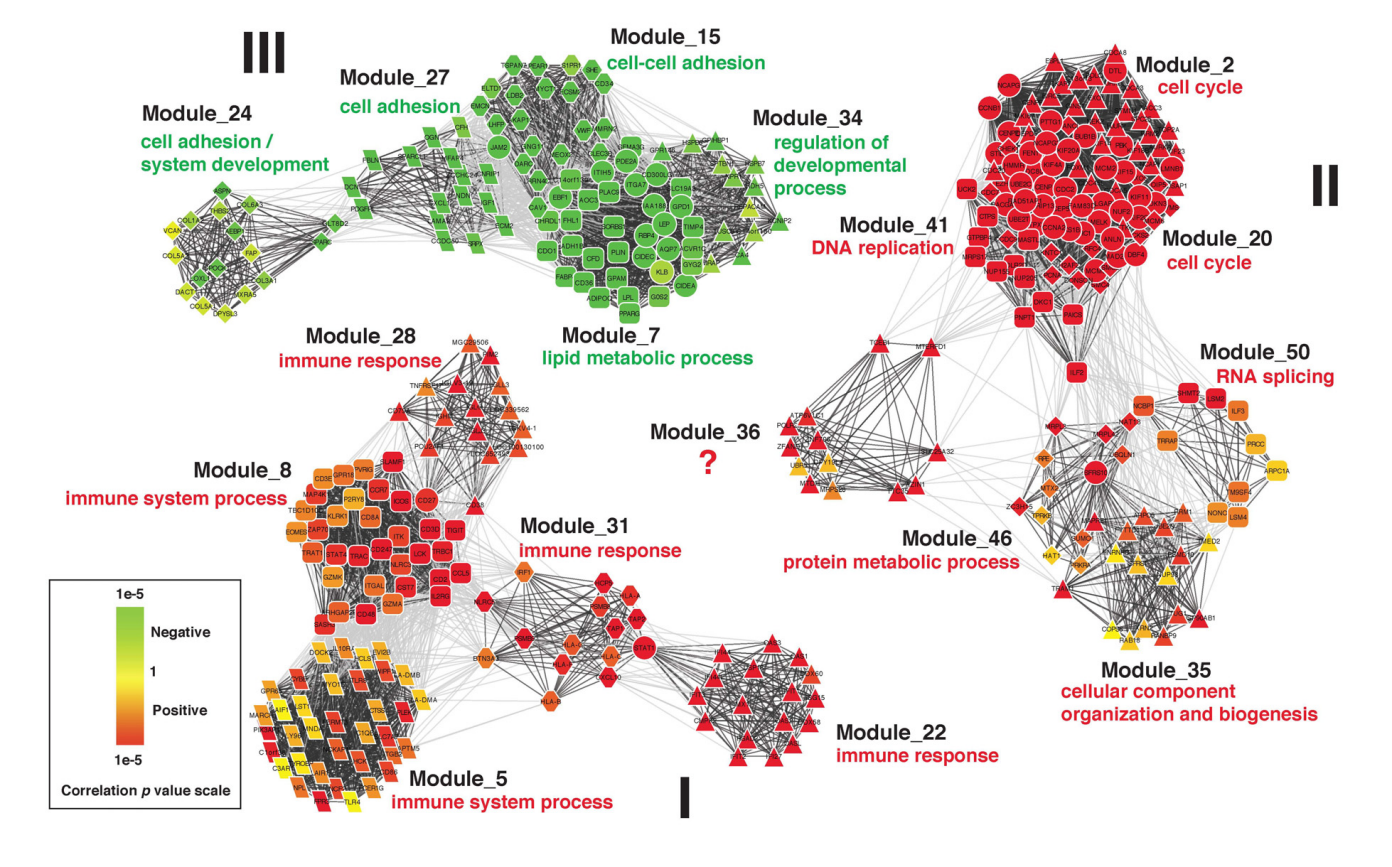
**Network of genes in the 17 modules reproducibly correlated with tumor grade**. Nodes represent genes and edges represent co-expression between genes. Edges connecting genes in the same module are colored in dark grey, while edges connecting genes in different modules are colored in light grey. Genes in the same module are represented by nodes of the same shape. Genes belonging to multiple modules are represented by round nodes. The color scale bar shows the scale of *p *values for correlations between gene expression and tumor grade as calculated by the Jonckheere-Terpstra test. Modules are clustered into three connected components I, II, and III. Primary function annotations for each module are labeled in the figure.

To understand the biology related to the grade-correlated modules and the interaction among the modules, we created a network for genes within the 17 reproducible ICE modules based on the GSE2109_breast dataset. As shown in Figure 4, the modules can be divided into three major groups, corresponding to the three connected components in the figure.

Group I included five up-regulated modules, all of them were related to immune system processes or immune response. Group II included seven up-regulated modules, six of which were related to cell proliferation. Group III included five down-regulated modules that were involved in cell adhesion and systems development. High cell proliferation in high-grade tumors fits with a 'hallmark' of cancer being uncontrolled proliferation [33]. Low cell adhesion levels in high-grade tumors is consistent with the fact that the loss of cell adhesion is required for cells to invade tissues and reach the bloodstream, where they can travel to distant sites to establish metastases [34]. Although the exact roles of the immune system during cancer development is complex and still a matter of debate [35], the association between immune system and cancer development has been known for over a century [36]. These results demonstrate that the module-based approach provides easily interpretable characterization of transcriptional changes. By correlating modules instead of individual genes with tumor grades, we gain a higher-order understanding of the biological processes related to breast cancer progression.

Although the ICE algorithm aims at identifying relatively independent modules by ensuring a proportion of unique genes in individual modules (10 in this study), we noticed considerable overlap among some of the modules (e.g. Module_2 and Module_20). It is not clear whether these modules are really independent. However, downstream transcription factor target enrichment analysis could help elucidate whether they are associated with different regulatory mechanisms.

### Genomic gain during breast tumor progression

Module_36 in Group II (Figure 4) was not enriched in any GO categories and would not have been identified using any knowledge-driven analyses based on GO. However, careful examination of the module found that all 13 genes in this module lie in close proximity on chromosome 8, suggesting potential biological relevance of the module. Specifically, 9 of the genes are located in the 8q22 region, 3 in 8q21, and 1 in 8q23 (Figure 4). If we randomly pick 13 genes from all 19,803 genes on the microarray, we only expect to find 0.09 genes in the 8q21-23 region that has a total of 144 genes. Finding all 13 genes from the module in this region is significantly non-random (*p *= 2.92e-28 in the Hypergeometric test).

**Figure 5:**
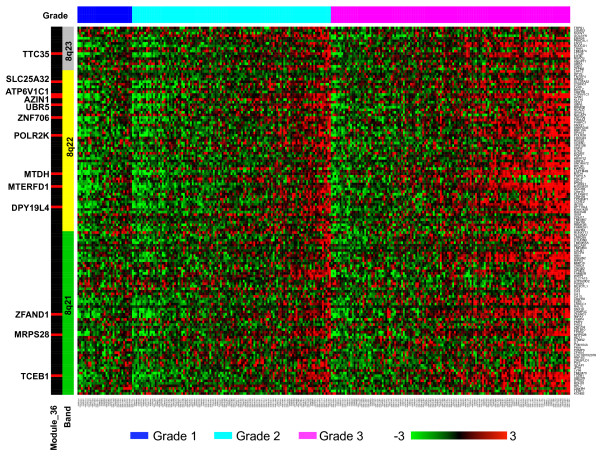
**Expression profile of genes in the chromosome region 8q21-23**. Expression data for genes in the chromosome region 8q21-23 were collected from the GSE2109 dataset and visualized in a heat map. Rows present genes and columns represent samples. Genes are ordered by their chromosome location and colored-coded on the left by three cytogenetic bands 8q21, 8q22, and 8q23. Genes in module_36 are marked in red and labeled on the left. Samples are color-coded on the top by tumor grade, where blue, cyan, and pink correspond to grades 1, 2, and 3, respectively. The color scale bar at the bottom shows the relative gene expression level (0 is the mean expression level of a given gene).

To check whether this enrichment was due to the genomic gain of 8q21-23 in high-grade tumors, we plotted the gene expression data for all genes in this region based on the GSE2109_breast dataset (Figure 5). It is clear from the figure that not only the 13 genes, the majority of genes in this region showed consistent and elevated expression in high-grade tumors. Previously, genomic gain at 8q22 in breast tumor samples has been reported by independent groups and has been associated with poor-prognosis [37,38]. A recent study identified MTDH, one gene in module_36, as the most significant functional mediator of this poor-prognosis genomic gain [37]. Our results indicate that the genomic gain may affect a broader region including 8q21, 8q22, and 8q23. Interestingly, a published bioinformatics study using 12 independent human breast cancer microarray studies comprising 1422 tumor samples also identified 8q21-23 as a potential aberrant chromosomal region [39]. It is not clear why this region is consistently duplicated in high-grade tumors. However, because Module_36 is moderately correlated with the cell proliferation modules (Figure 4), we hypothesize that this genomic instability may be associated with increased cell proliferation.

### Regulatory mechanisms underpinning breast tumor progression

**Figure 6:**
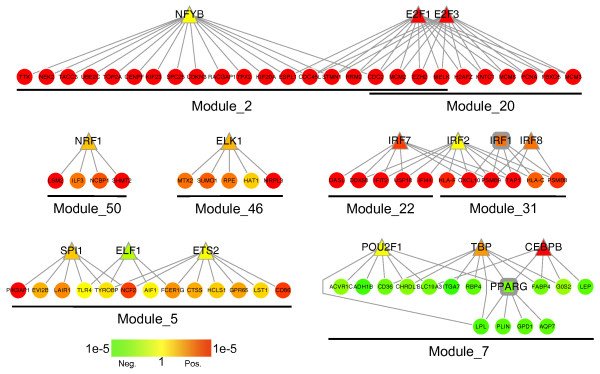
**Co-expression modules and their regulators**. Nodes represent genes and edges represent transcriptional regulation between transcription factors (top) and target genes (bottom). Transcription factors that are also included in the module are represented by square nodes, while others are represented by triangle nodes. The color scale bar shows the scale of *p *values for correlations between gene expression and tumor grade as calculated by the Jonckheere-Terpstra test.

Because genes in a co-expression module are likely to be regulated by a cohesive mechanism [8], we performed the functional category enrichment analysis for the modules against the gene sets of transcription factor targets in order to identify potential transcriptional regulatory mechanisms underlying the modules. Among the 17 modules, 8 were enriched with targets of certain transcription factors (B-adjp less than 0.05, Figure 6).

In a previous study using a different data set (GSE3494), Niida *et al. *employed a Bayesian network approach and identified motifs bound by ELK1, E2F, NRF1 and NFY as principal regulatory motifs driving malignant progression of breast cancer [40]. All these transcription factors were detected in our study. Genes regulated by NYFB, E2F1/E2F3, NRF1, and ELK1 were enriched in module_2, module_20, module_50, and module_46, respectively. All these previously reported regulatory mechanisms are related to increased cell proliferation in high-grade tumors. The result that overlapping modules such as module_2 and module_50 are associated with different transcription factors (Figure 6) suggests that they are likely to be independent.

We also identified regulatory mechanisms related to increased immune response in high-grade tumors. Specifically, transcription factors in the IRF family (IRF1, IRF2, IRF7, and IRF8) were associated with module_22 and module_31, and those in the ETS family (SPI1, ELF1, and ETS2) were associated with module_5. Both of these families have implicated roles in breast cancer [41,42].

Module_7 was down-regulated in high-grade tumors, and it was enriched with the targets of transcription factors CEBPB, TBP, POU2F1, and PPARG. Interestingly, PPARG itself was included in the module and is a target of all the other three transcription factors. Eight out of the 35 genes in this module, including LPL, SORBS1, PPARG, PLIN, FABP4, AQP7, CD36, and ADIPOQ, are involved in the PPARA signaling pathway according to the annotation in the KEGG database. The result indicates that this transcriptional program might contribute to breast tumor progression through inhibiting the PPARA signaling pathway. Consistently, it has been recently reported that the odds of breast cancer are doubled among women with PPARA polymorphism rs4253760 [43].

As shown in Figure 6, some transcription factors, such as IRF1 and PPARG are highly correlated with target genes and therefore are included in the modules. Some transcription factors are not included in the module but still show positive correlation with target genes, such as E2F1, E2F3, and IRF7. Some transcription factors are negatively correlated with target genes such as CEBPB. It is worth noting that most of the transcription factors do not show significant transcriptional changes and cannot be identified by individual gene based analysis. Our module-based approach not only confirmed previously reported regulatory mechanisms, but also generated novel hypotheses on regulatory mechanisms associated with breast tumor progression.

### Prognostic value of the grade-correlated modules

It is well known that morphologically similar tumors may have very different clinical courses. Because tumor specimens with the same grade annotation showed very different expression pattern of the modules (see Additional file 1), we explored whether expression pattern of the modules could be used to classify tumors more accurately with regard to their likelihood of recurrence. Specifically, we applied unsupervised hierarchical clustering on the GSE2990 dataset to cluster cancer patients into subgroups based on the expression pattern of the 17 modules, and then investigated difference in relapse-free survival for different patient subgroups by comparing their survival curves estimated using the Kaplan-Meier method.

**Figure 7:**
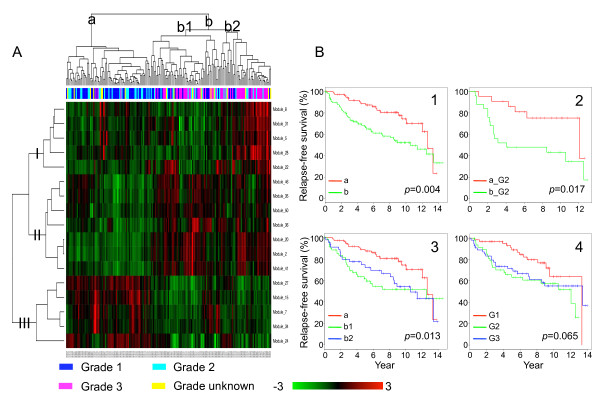
**Patient subgroups identified based on module expression pattern and their relapse-free survival**. Panel A: two-dimensional clustering of the samples and the 17 modules in the GSE2990 dataset. Rows represent modules and columns represent samples, which are colored-coded by tumor grade, where blue, cyan, pink, and yellow correspond to grades 1, 2, 3, and unknown, respectively. Module expression is calculated as the average standardized expression of all genes in the module. The color scale bar shows the relative module expression level, where 0 is the mean expression level of a given module. Modules and samples are clustered independently by hierarchical clustering. Major module clusters (I, II, and III) and major patient clusters (a, b1, and b2) are labeled on the dendrograms. Panel B shows the survival analysis results for different patient subgroups. B1: group a versus group b, all tumor grades. B2: group a versus group b, limited to grade 2 (G2) tumors. B3: groups a, b1, and b2, all tumor grades. B4: grade 1(G1), 2 (G2), and 3 (G3).

As shown in Figure 7A, the modules can be categorized into 3 groups based on the overall expression patterns, corresponding to the three connected components in Figure 4. Patients in the dataset are first divided into two groups based primarily on the expression of group II and group III modules. The group with low-expression of group II modules and high-expression of group III modules (patient group a) comprised mostly patients with grade 1 or 2 tumors, while the group with high-expression of group II modules and low-expression of group III modules (patient group b) was enriched in patients with grade 3 tumors. This was expected as all of the modules were significantly correlated with tumor grade in this dataset, although they were identified in the independent GSE2109_breast dataset. Patient group b can be further divided into two subgroups based on the expression of group I modules. One subgroup showed low-expression of group I modules (patient group b1), while the other showed high-expression of these modules (patient group b2).

We first investigated whether the patient groups a and b had different recurrences. As shown in Figure 7B_1, they did show significant different relapse-free survival (*p *= 0.004 in log-rank test). To test whether this result was independent of tumor grade, we limited the survival analysis to grade 2 patients. As shown in Figure 7B_2, grade 2 patients in groups a and b also showed significant different relapse-free survival (*p *= 0.017), even with a small sample size.

Next, we compared relapse-free survival for patient groups a, b1, and b2 as identified from the hierarchical clustering. According to the log-rank test, the overall difference among these groups was statistically significant (*p *= 0.013). As shown in Figure 7B_3, group a patients had obviously better outcome than group b patients. Moreover, outcomes for the two subgroups in group b were also discernible: patients in group b1 seemed to have higher risk of recurrence compared to those in group b2. Considering the function of the modules (Figure 4), the result suggests that patients with lower cell proliferation and higher systems development and cell adhesion have lower risk of recurrence. For patients with high cell proliferation, although the sample size was too small to draw any statistical conclusions, it seems that the subgroup with higher immune response has relatively lower risk of recurrence, at least from year 4 to year 8. One explanation is that the activation of adaptive immunity in the high cell proliferation group could elicit antitumor responses through T-cell-mediated toxicity, antibody-dependent cell-mediated cytotoxicity, and antibody-induced complement-mediated lysis. However, as immune system may play paradoxical roles during cancer development[35], the relationship between immune system activity and the risk of recurrence may not be straightforward.

In comparison, we investigated the association between grade annotation and relapse-free survival. As shown in Figure 7B_4, grade 1 patients had considerable better outcome than grade 2 and 3 patients, while the outcomes for grade 2 and 3 patients were hardly discernible. The overall difference in relapse-free survival for the three groups with different tumor grades was only marginally significant (*p *= 0.065).

These results demonstrate that the expression pattern of the grade-correlated modules is associated with the risk of recurrence, that the expression pattern of the modules can classify grade 2 patients into two groups with high versus low risks of recurrence, and that the three patient groups identified by the module expression pattern may classify patients more accurately than the traditional grading system. Similar findings have been reported in the original study in which a gene-level approach was applied on the GSE2990 dataset [28]. However, our module-based approach puts genes into biological contexts and helps reveal biological processes and regulatory mechanisms underlying the biomarkers. Along the same line, protein interaction modules have been proposed as biomarkers for the classification of breast cancer metastasis[44]. As co-expression modules are sub-networks identified from gene co-expression networks in a data-driven fashion, they are not limited by existing knowledge on protein interaction and should compliment the protein interaction module-based approach. An obvious extension of the current study is to construct prediction models based on the co-expression modules and to evaluate their performance in different patient cohorts.

## Conclusions

We have developed a novel algorithm ICE for identifying relatively independent maximal cliques as co-expression modules and a co-expression module-based approach to the analysis of gene expression data. The ICE algorithm can identify functionally homogeneous modules from complex gene co-expression networks. Because the overlap among the modules is restrained, the algorithm is able to identify a small number of modules covering a variety of biological processes, in turn facilitating downstream analyses to investigate major transcriptional programs encoded in gene co-expression networks. On the other hand, the stringent requirement imposed by the module definition in the algorithm ensures high-level of co-expression among all genes in a module. Therefore, the algorithm is able to identify truly co-regulated gene sets that are reproducible in independent data sets. Applying the module-based approach on a breast cancer microarray gene expression dataset revealed important biological processes and previously reported regulatory mechanisms underlying breast tumor progression. In addition, novel hypotheses on regulatory mechanisms and genomic gain associated with breast tumor progression have been generated. The 17-module signature of breast tumor progression clustered patients into subgroups with significantly different relapse-free survival times in an independent dataset and provided useful prognostic information that is independent of tumor grade.

## Methods

### Data preprocessing

cel files for each gene expression dataset were downloaded from the Gene Expression Omnibus (GEO) database http://www.ncbi.nlm.nih.gov/geo/ and processed using the Robust MultiChip Analysis (RMA) algorithm [45] as implemented in *Bioconductor *[46]. Probe set identifiers (IDs) were mapped to gene symbols based on the mapping provided by the GEO database. Median expression levels from multiple probe sets corresponding to the same gene were calculated to represent the gene expression level. To make the gene expression level comparable across genes, expression values for each gene were standardized using a Z-score transformation. For each dataset, a gene expression matrix with normalized and standardized expression values was thus generated.

### Gene co-expression network construction

Given a gene expression matrix with *n *genes and *m *samples, Pearson's correlation coefficients are calculated for all the  gene pairs. To evaluate functional similarity between each pair of genes, the Resnik's semantic similarity [47] is calculated based on the GO biological process annotation according to Elo et al [25]. The average functional similarity of gene pairs at various correlation ranges is calculated and plotted. A correlation threshold above which a sharp increase in functional similarity occurs is selected through manual inspection of the plot. Based on the selected threshold, a gene co-expression network is constructed in which each node is a gene while two nodes are connected by an edge if their Pearson's correlation coefficient is above the threshold.

### Co-expression module identification

We propose an intuitive Iterative Clique Enumeration (ICE) algorithm to identify a manageable number of maximal cliques as relatively independent co-expression modules in order to facilitate further analyses of the transcriptional mechanisms encoded in a gene co-expression network. Given a graph , *G *= (*V,E*), the algorithm works on the original graph *G *and "residual graphs" *G*_*i*_= (*V*_*i*_, *E*_*i*_) iteratively, where the residual graph *G*_*i *_is generated by removing all maximal cliques identified before step *i *from *G*. At step *i*, the algorithm first identifies the maximum clique *C*_*i *_in *G*_*i *_[48], and then finds the largest maximal clique *C*_*i*_^' ^in *G *that covers *C*_*i*_. *C*_*i*_*'* is considered as a module and is removed from *G*_*i *_to generate the next residual graph *G*_*i+1*_. Because *C*_*i *_is identified in a residual graph, it has no overlap with previously found modules. However, expanding *C*_*i *_to *C*_*i*_^' ^allows overlap between *C*_*i*_^' ^and existing modules. Therefore, overlap among the identified modules is allowed but the amount of overlap is restrained. The iteration stops when a pre-defined clique size threshold *c*_*min *_is reached. Because we focus on the major regulatory programs encoded in the network, *c*_*min *_is set to 10 in this study. The ICE algorithm is formally described in the Appendix. A C implementation is available at http://bioinfo.vanderbilt.edu/ice. For comparison, we included the popular K-means clustering algorithm and a graph-based module identification algorithm MCODE in the study. K-means clustering was performed on the gene expression matrix using the *kmeans *function in R. MCODE analysis was performed in Cytoscape [49]. Default parameters were used in both cases.

### Functional category enrichment analysis

For a module with *n *genes and an *a priori *defined functional category with *K *genes, hypergeometric test[50] is used to evaluate the significance of the overlap *k *between the module and the category. All *N *genes on the microarray are used as a reference. The significance of the overlap can be calculated by . For the source of the functional categories, we used the Gene Ontology (GO) gene sets and the transcription factor targets gene sets downloaded from the MSigDB (http://www.broad.mit.edu/gsea/msigdb/index.jsp, version 2.5). The later was organized by transcription factor binding motifs. We further collected known transcription factors for the binding motifs from the Transfac database (http://www.gene-regulation.com, professional version 12.1). Genes associated with different binding motifs that correspond to a common transcription factor were combined into one gene set. Gene sets associated with binding motifs that have no known transcription factors were not considered in this study.

### Overall expression level of a module

Because the gene expression data is normalized and standardized, for a selected sample and a specific module *M*_*j*_, average expression of all genes in the module is used to represent the overall expression of the module in the sample: .

### Jonckheere-Terpstra test

Suppose that *N *samples (e.g., tumor specimens) are assigned to *s *ordered categories (e.g., tumor grades) and a selected attribute *X *(e.g., expression of a gene) is measured for the samples and ranked. The Jonckheere-Terpstra test[51] tests the null hypothesis *H *that there are no difference among the categories. The alternative hypothesis is that the sample means change monotonically along the ordered sequence of the categories. Let us denote by *X*_*i1*_, ..., *X*_*im *_and *X*_*j1*_, ..., *X*_*jn *_the observations on the *i*th and *j*th categories where *i *<*j*, and by *W*_*ij *_the number of pairs (*α,β*) for which *X*_*iα *_<*X*_*jβ*_. The Jonckheere-Terpstra test statistic *W *can be calculated as . Based on the limit theorem, for large values of *N*, the statistic *W *is approximately normally distributed, with an expected mean of  and a variance of [51]. Therefore, the significance of *W *can be calculated based on the null distribution.

### Survival analysis

Survival analysis was performed in *R *using the *survival *package. Specifically, survival curves were estimated using the Kaplan-Meier method, and survival comparisons among groups were made by the log-rank test.

### Network visualization

Networks were visualized using Cytoscape [49].

## Authors' contributions

BZ and ZS conceived of the study, designed the algorithm, and performed the data analysis. BZ and CKD performed the data interpretation. BZ, ZS and CKD drafted the manuscript. All authors contributed to and approved the final manuscript.

## Appendix

Algorithm 1. Iterative Clique Enumeration (ICE)

1. Given graph *G *= (*V,E*), clique size threshold *c*_min_

2. *i *= 0, *C*_0 _is the maximum clique in *G*, output *C*_0_

3. *C*_0_^' ^← *C*_0_, *G*_0 _← *G*, *V*_0 _← *V*, *E*_0 _← *E*

4. while ||*C*_*i*_|| ≥ *c*_min _do

5. *i *← *i* + 1

6. *G*_*i *_= (*V*_*i*_, *E*_*i*_), where 

7. Find the maximum clique *C*_*i *_in *G*_*i*_

8. Find the largest maximal clique *C*_*i*_' in *G *such that *C*_*i*_' ⊇ *C*_*i*_

9. Output *C*_*i*_'

10. end while

## Supplementary Material

Additional file 1 **Dynamic expression of the modules during breast cancer progression**. Figures visualizing the dynamic expression of the modules during breast cancer progression in the GSE2109 dataset. Click here for file
