# Supplementary material for: Co-expression module analysis reveals biological processes, genomic gain, and regulatory mechanisms associated with breast cancer progression
Source: BMC Syst Biol. 2010 May 27;4:74. doi: 10.1186/1752-0509-4-74 (PMC2902438; doi:10.1186/1752-0509-4-74)

Additional File 1. Dynamic expression of the modules during breast cancer progression. Panel A shows the expression profiles of the modules in the GSE2109 dataset. Rows represent modules and columns represent samples, which are colored-coded by tumor grade, where blue, cyan, and pink correspond to grades 1, 2, and 3, respectively. Module expression is calculated as the average standardized expression of all genes in the module. The color scale bar shows the relative module expression level (0 is the mean expression level of a given module). Samples are grouped by tumor grade, and then clustered based on the module expression patterns within the same grade. Modules are clustered by hierarchical clustering. Three main module clusters (I, II and III) are labeled on the dendrogram, corresponding to the connected components in Figures 3. Panel B shows the expression trend for individual modules during tumor progression. Modules are grouped for presentation based on the three main clusters as indicated in Panel A.

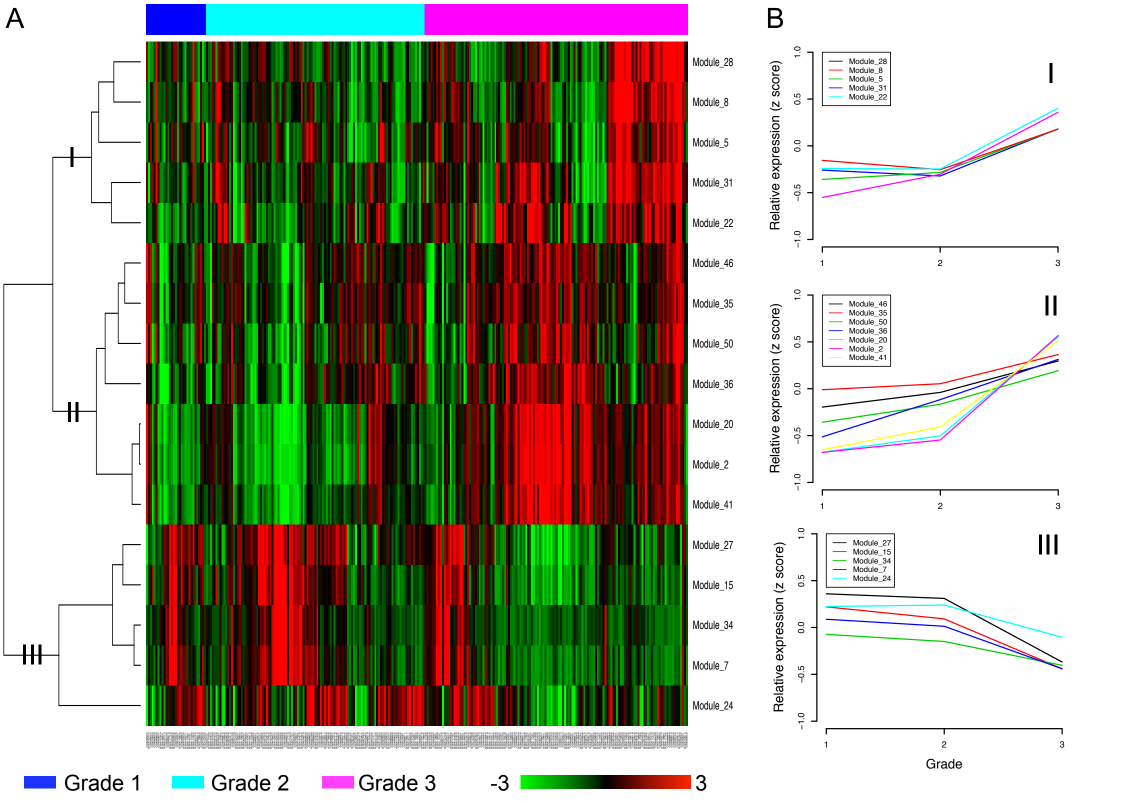

Supplement: Additional file 1 — Dynamic expression of the modules during breast cancer progression. Figures visualizing the dynamic expression of the modules during breast cancer progression in the GSE2109 dataset. [file 1752-0509-4-74-S1.PDF]
